# The relationship between arterial transducer level and pulse contour waveform-derived measurements

**DOI:** 10.1186/s13054-015-0745-8

**Published:** 2015-02-02

**Authors:** Huaiwu He, Dawei Liu, Yun Long, Xiaoting Wang, Yuan Yu, Xin Li, Hailing Guo, Jing Cai, Ning Fang

**Affiliations:** Department of Critical Care Medicine, Peking Union Medical College Hospital, Chinese Academy of Medical Science, 1 shuaifuyuan, Dongcheng District, Beijing, 100730 China

**Keywords:** ■■■

For hemodynamic monitoring, the pressure transducer is suggested to be fixed at the level of the phlebostatic axis in critically ill patients [1,2]. The correction and adjustment of pressure transducer are emphasized in central venous pressure monitoring in clinical practice. The exact position of the transducer is relatively easy to be ignored for invasive arterial blood pressure monitoring [3,4]. Improper position of the transducer may cause inaccurate value and shape of the arterial blood pressure wave, which would result in an invalid PiCCO (Pulsion Medical Systems AG, Munich, Germany) algorithm for pulse contour waveform-derived measurements. This study was conducted as a prospective quantitative evaluation of the relationship between arterial transducer level and pulse contour waveform-derived measurements.

In total, 22 patients were enrolled in the 28-bed department of critical care medicine of a university hospital. All of the patients had a femoral artery catheter for PiCCO hemodynamic monitoring. The site of the phlebostatic axis was defined as the zero level (reference level). We moved the arterial pressure transducer up and down at eight different levels (−5 cm, −10 cm, −15 cm, −20 cm, 5 cm, 10 cm, 15 cm, 20 cm). At each level, continuous cardiac index (CCI), rate of left ventricular pressure rise during systole (dP/dtmax), and systemic vascular resistance index (SVRI) were simultaneously recorded.

The elevation of pressure transducer caused significantly positive changes in CCI and negative changes in dP/dtmax and SVRI, which resulted in a change in the opposite direction for these parameters (−CCI, +dP/dtmax, and + SVRI). When the change of the transducer’s position was 5 cm, the changes of SVRI with error reached statistical significance (*P* < 0.0001), but there were no differences in the CCI between the 5 cm and 0 cm reference level (CCI: +5 cm versus 0 cm 3.2 ± 0.6 versus 3.2 ± 0.7, *P* = 0.715; −5 cm versus 0 cm 3.1 ± 0.7 versus 3.2 ± 0.7, *P* = 0.075). When the variation of transducer level was 10 cm, the change of CCI with error reached statistical significance (*P* < 0.0001) (Figure 1). Different positions of the transducer and the corresponding changes in CCI and dP/dtmax are shown in Table 1. Figure 2 shows the trends of estimated marginal means of CCI, CCI change rate, dP/dtmax, and dP/dtmax change rate at different positions of the transducer.

**Figure 1:**
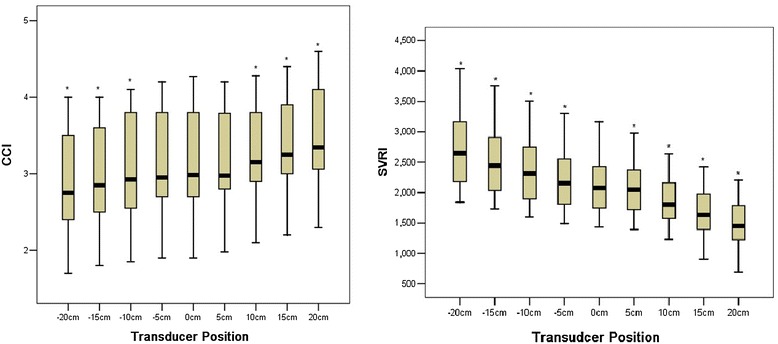
**The variation of continuous cardiac index (CCI) and systemic vascular resistance index (SVRI) at different levels of the pressure transducer.** *P < 0.0001 versus 0 cm transducer position. CCI is presented as liters per minute per square meter, and SVRI is presented as dyne per second per centimeter raised to the fifth power.

**Table 1:** **Corresponding vertical distances and the changes and variation rates of continuous cardiac index changes and dP/dtmax**

**Vertical distance**	**CCI change, L/minute per m** ^**2**^	**Rate of CCI change, percentage**	**dP/dtmax change, mm Hg/second**	**Rate of dP/dtmax change, percentage**
+20 cm	0.36	11.9	−36	−3.5
+15 cm	0.24	7.9	−27	−2.6
+10 cm	0.13	4.5	−17	−1.6
+5 cm	0.01	0.31	−6	−0.65
−5 cm	−0.03	−0.85	9	0.9
−10 cm	−0.11	−3.4	21	2.0
−15 cm	−0.20	−6.4	32	3.1
−20 cm	−0.29	−9.3	44	4.1

Data are presented as the mean. CCI, continuous cardiac index; dP/dtmax, rate of left ventricular pressure rise during systole.

**Figure 2:**
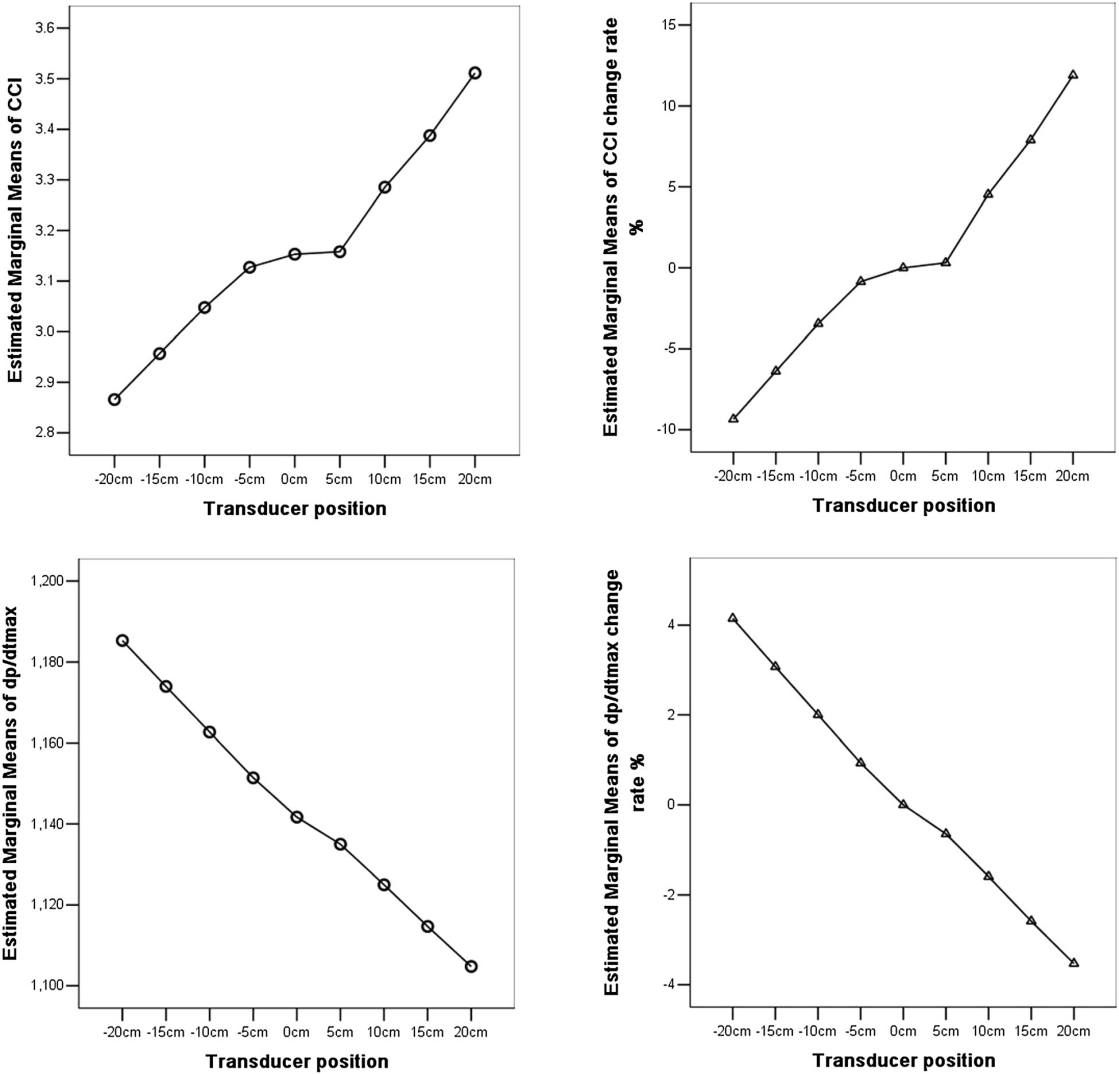
**The variation of estimated marginal means of continuous cardiac index (CCI), CCI change rate, rate of left ventricular pressure rise during systole (dP/dtmax), and dP/dtmax change rate.**

The contour waveform-derived parameter response to the change transducer is still undetermined. To the best of our knowledge, this is the first study to explore the impact of arterial transducer level on pulse contour waveform-derived parameters in clinical practice. The clinical implications of such errors are important to recognize. Our study provided evidence that the arterial transducer should be considered in order to obtain precise pulse contour waveform-derived parameters, especially when the transducer’s vertical distance was more than 10 cm from the phlebostatic axis. We believe that these findings deserve emphasis and should be applied in continuous hemodynamic monitoring.

## Abbreviations

CCI: Continuous cardiac index
dP/dtmax: rate of left ventricular pressure rise during systole
SVRI: Systemic vascular resistance index
